# Molecular Epidemiology and Genetic Diversity of Multidrug-Resistant Mycobacterium tuberculosis Isolates in Bangladesh

**DOI:** 10.1128/spectrum.01848-21

**Published:** 2022-02-23

**Authors:** S. M. Mazidur Rahman, Arfatur Rahman, Rumana Nasrin, Md. Fahim Ather, Sara Sabrina Ferdous, Shahriar Ahmed, Mohammad Khaja Mafij Uddin, Razia Khatun, Mohammad Shahnewaz Sarker, Asif Mujtaba Mahmud, Md. Mojibur Rahman, Sayera Banu

**Affiliations:** a Infectious Diseases Division, icddr,b, Dhaka, Bangladesh; b Medicinal Chemistry, Monash Institute of Pharmaceutical Sciences, Monash University, Parkville, Victoria, Australia; c Department of Respiratory Medicine, Asgor Ali Hospital, Gandaria, Dhaka, Bangladesh; d Department of Epidemiology, Bangladesh University of Health Sciences, Darus Salam, Mirpur, Dhaka, Bangladesh; Johns Hopkins University School of Medicine

**Keywords:** Bangladesh, MDR-TB, MIRU-VNTR, *Mycobacterium tuberculosis*, molecular epidemiology, spoligotyping

## Abstract

Although the number of multidrug-resistant (MDR) tuberculosis (TB) cases is high overall, a major gap exists in our understanding of the molecular characteristics and transmission dynamics of the MDR Mycobacterium tuberculosis isolates circulating in Bangladesh. The present study aims to characterize the MDR-TB isolates of Bangladesh and to investigate the mode of transmission. A total of 544 MDR-TB isolates were obtained from a nationwide drug-resistant TB surveillance study conducted between October 2011 and March 2017 covering all geographic divisions of Bangladesh. The isolates were characterized using TbD1 deletion analysis, spoligotyping, and mycobacterial interspersed repetitive-unit–variable-number tandem-repeat (MIRU-VNTR) typing. Deletion analysis showed that 440 (80.9%) isolates were the modern type, while the remainder were the ancestral type. The largest circulating lineage was the Beijing type, comprising 208 isolates (38.2%), followed by T, EAI, and LAM with 93 (17.1%), 58 (10.7%), and 52 (9.5%) isolates, respectively. Combined MIRU-VNTR and spoligotyping analysis demonstrated that the majority of the clustered isolates were of the Beijing and T1 lineages. The overall rate of recent transmission was estimated at 33.8%. In conclusion, the MDR M. tuberculosis isolates circulating in Bangladesh are mostly of the modern virulent type. The Beijing and T lineages are the predominant types and most of the transmission of MDR-TB can be attributed to them. The findings also suggest that, along with the remarkable transmission, the emergence of MDR-TB in Bangladesh is largely due to acquired resistance. Rapid and accurate diagnosis and successful treatment will be crucial for controlling MDR-TB in Bangladesh.

**IMPORTANCE** Multidrug-resistant TB is considered to be the major threat to tuberculosis control activities worldwide, including in Bangladesh. Despite the fact that the number of MDR-TB cases is high, a major gap exists in our understanding of the molecular epidemiology of the MDR-TB isolates in Bangladesh. In our study, we characterized and classified the MDR-TB isolates circulating in Bangladesh and investigated their mode of transmission. Our results demonstrated that the MDR M. tuberculosis isolates circulating in Bangladesh are mostly of the modern virulent type. The Beijing and T lineages are the predominant types and are implicated in the majority of MDR-TB transmission. Our findings reveal that, along with the remarkable transmission, the emergence of MDR-TB in Bangladesh is largely due to acquired resistance, which may be due to nonadherence to treatment or inadequate treatment of TB patients. Rapid diagnosis and adherence to an appropriate treatment regimen are therefore crucial to controlling MDR-TB in Bangladesh.

## INTRODUCTION

Tuberculosis (TB) is an airborne infectious disease caused by Mycobacterium tuberculosis, which alongside the human immunodeficiency virus (HIV), is the leading cause of death worldwide (1). Multidrug-resistant TB (MDR-TB), caused by strains that are simultaneously resistant to isoniazid (INH) and rifampicin (RIF), is the major threat to TB control activities across the globe (2). In 2019, an estimated 10.0 million people developed TB worldwide, an estimated half a million of whom had MDR-TB. Bangladesh is one of the countries with a high burden of both TB and MDR-TB, as an estimated 47,000 people in Bangladesh die due to TB each year. According to a recent drug resistance survey, the burden of MDR-TB has shown a downward trend in new (0.7% versus 1.4%) and previously treated patients (11% versus 28.5%) (1). Regardless of the improvements in strategies designed to prevent, diagnose, and treat TB, only around half (52 to 56%) of the world’s MDR-TB patients are treated successfully. The poor outcome of this treatment leads to increased mortality and a higher chance of developing a severe form of extensively drug-resistant TB (XDR-TB) (1, 2).

Rapid detection, adequate therapy, and contact tracing are the key components of properly managing TB and impeding further transmission in the community. Particularly virulent strains are expanding aggressively in some communities or geographical areas; for example, strains belonging to the Beijing family have shown an association with increased drug resistance and outbreaks of MDR-TB in many Asian countries (3–5). A previous genotypic study in Bangladesh has demonstrated the prevalence of Beijing strains in this country (6). One of our previous studies in the rural community of Matlab demonstrated that TB in rural Bangladesh is caused primarily by the reactivation of latent infections, along with the recent emergence of Beijing strain clusters (7). Most of the members of the Beijing family are resistant to many first- and second-line drugs used in TB treatment. The emergence of multiple variants of the Beijing lineage has resulted in increased transmissibility and drug resistance (8). It is therefore very important to rapidly identify these genotypes, as this will facilitate the adoption of appropriate control measures to stop further transmission.

The genotyping of mycobacterial strains using molecular methods has improved the knowledge and understanding of TB epidemiology, along with its transmission dynamics, and has further become an important tool for molecular-guided TB surveillance, control, and prevention (5, 9). Spoligotyping and mycobacterial interspersed repetitive-unit–variable-number tandem-repeat (MIRU-VNTR) typing are the PCR-based methods most commonly used in recent molecular epidemiological studies of TB (7, 10, 11). Spoligotyping allows the simultaneous detection and typing of isolates belonging to the M. tuberculosis complex into various lineages and sublineages, while the MIRU-VNTR genotyping method is used for detailed cluster analysis (5, 10). Recently, an automated MIRU-VNTR typing method has been optimized using the QIAxcel advanced system, which reduces the associated costs and time; hence, it can be applied for a large number of samples simultaneously (12, 13). Deletion analysis is a novel PCR-based technique used to differentiate the members of the M. tuberculosis complex into “ancestral” or “modern” types based on the presence or absence of the TbD1 region in the genome (TbD1^+^ or TbD1^−^, respectively) (14). Studies have shown that many modern-type strains were associated with some of the major TB epidemics (14).

Even though the rates of MDR-TB are high, there are still major gaps in our knowledge of the molecular characteristics and transmission dynamics of MDR-TB isolates from all over Bangladesh. The present study aims to characterize and classify the MDR-TB isolates circulating in different areas of Bangladesh and investigate the mode of transmission using a combination of TbD1 deletion analysis, spoligotyping, and MIRU-VNTR typing techniques. Additionally, we aim to gain insights into any association between the genotypic characteristics of MDR-TB isolates and their drug resistance patterns.

## RESULTS

The MDR-TB isolates were obtained from a nationwide sentinel surveillance program aimed at detection of MDR and XDR-TB in Bangladesh that was conducted between the periods of 2011 and 2017. The MDR-TB isolates were characterized by using deletion analysis, spoligotyping, and MIRU-VNTR typing. The demographic and clinical characteristics, drug resistance patterns, geographical distribution, and transmission dynamics of MDR-TB strains were investigated.

### Demographic characteristics of the study participants.

A total of 544 MDR-TB patients were investigated in this study; of these, 65% (*n* = 353) were male and the remainder were female (Table 1). The median age of the patients was 34 years, with an interquartile range of 20 to 57 years, and 56.3% were aged between 21 and 40 years. About 83% of the patients had no previous exposure to other TB patients. Around 92% of the patients had a history of TB disease and had been treated with anti-TB drugs. Details of exposure and/or TB history, along with previous treatment outcomes, are summarized in Fig. S1 in the supplemental material. Treatment outcome data were available for 413 of 500 previously treated patients. An analysis found that about 41.6% (172/413) of patients had completed the treatment and/or been cured, while 58.4% (241/413) had experienced treatment regimen failure or did not complete the treatment (Fig. S1).

**TABLE 1:** Demographic and clinical characteristics of MDR-TB patients

Characteristic	Variable	No. of participants (*n* = 544)	Frequency (%)
Sex	Male	353	64.9
	Female	191	35.1
Age (yrs)	≤20	98	18.1
	21–40	306	56.3
	41–60	114	21.0
	>60	25	4.6
Smoking	Yes	148	27.2
	No	396	72.8
Drug user	Yes	16	2.9
	No	528	97.1
Dwelling	Rural	280	51.5
	Urban	264	48.5
Exposure to TB patients	Yes	93	17.1
	No	451	82.9
Previous history of TB	Yes	501	92.1
	No	43	7.9
Previous treatment history	Yes	500	91.9
	No	44	8.1
Geographic location	Chittagong	253	46.5
	Mymensingh	88	16.2
	Rajshahi	82	15.1
	Rangpur	75	13.8
	Dhaka	28	5.1
	Khulna	11	2.0
	Barishal and Sylhet	07	1.3

Specimens were collected from all eight geographical divisions of the country. Patients of rural and urban origin were almost equally distributed (51.5% versus 48.5%). Almost half of the patients (253; 46.5%) were from the Chittagong division, which is home to diverse ethnic groups, followed by 88 (16.2%), 82 (15.1%), and 75 (13.8%) from Mymensingh, Rajshahi, and Rangpur divisions, respectively. The rest of the MDR-TB patients (46; 8.4%) were from other geographical divisions, including Dhaka, Khulna, Barishal, and Sylhet (Table 1).

### Characteristics of strains based on deletion analysis.

All 544 isolates were confirmed as M. tuberculosis based on the presence of the RD9 region. Strains were analyzed by PCR for the presence or absence of the TbD1 region. Deletion analysis revealed that the TbD1 region was absent in 440 (80.9%) isolates, indicating that these isolates correspond to the modern type (Table 2). In contrast, the TbD1 region was intact in only 104 (19.1%) isolates, which were thus deemed to belong to the ancestral type.

**TABLE 2:** Genotypes of MDR M. tuberculosis strains based on deletion analysis and spoligotyping

Category	No. of isolates (*n* = 544)	% of isolates
TbD1 type		
Modern (TbD1^−^)	440	80.9
Ancestral (TbD1^+^)	104	19.1

Spoligotype (lineage)		
Beijing	208	38.2
T	93	17.1
EAI	58	10.7
LAM	52	9.5
CAS	49	9.1
H	5	0.9
S	2	0.4
Manu	1	0.2
AFRI	1	0.2
New/undefined (with SIT)	9	1.6
Orphan	66	12.1

### Genotype determination by spoligotyping.

Based on the SITVIT2 database, spoligotyping of 544 isolates revealed 10 broad lineages comprising 478 (87.9%) isolates covering 61 different spoligotype international types (SITs) (Table 2). There were 66 (12.1%) isolates for which specific lineages could not be determined; these were classified as “Orphan.” The largest lineage was the Beijing type, comprising 208 isolates (38.2%), followed by T, EAI, LAM, and CAS, with 93 (17.1%), 58 (10.7%), 52 (9.5%), and 49 (9.1%) isolates, respectively (Table 2). Lineages with very few isolates, such as H (5), S (2), Manu (1), and AFRI (1), were also identified. Additionally, we found nine strains with SIT2152 and 1,089 without any defined lineage that were categorized as “new.”

Based on the specific SIT numbers, the broad lineages were divided into defined sublineages (Table S1). Among the Beijing isolates, 203 were classical type (SIT1) and 5 were a nonclassical (SIT265, SIT269, SIT796, or SIT2610) type. The common sublineages of T-type strains were T1, with SIT53 (*n* = 38), SIT123 (*n* = 10), SIT244 (*n* = 19), and SIT358 (*n* = 17). Similarly, the most common sublineages of the LAM and CAS families were LAM9 (SIT42; *n* = 43) and CAS1-Delhi (SIT26; *n* = 36), respectively. In contrast, the EAI lineage was very diverse and divided into 25 different SITs covering sublineages of EAI1-SOM, EAI3-IND, EAI5, EAI6-BGD, and EAI7-BGD (Table S1).

**FIG 1:**
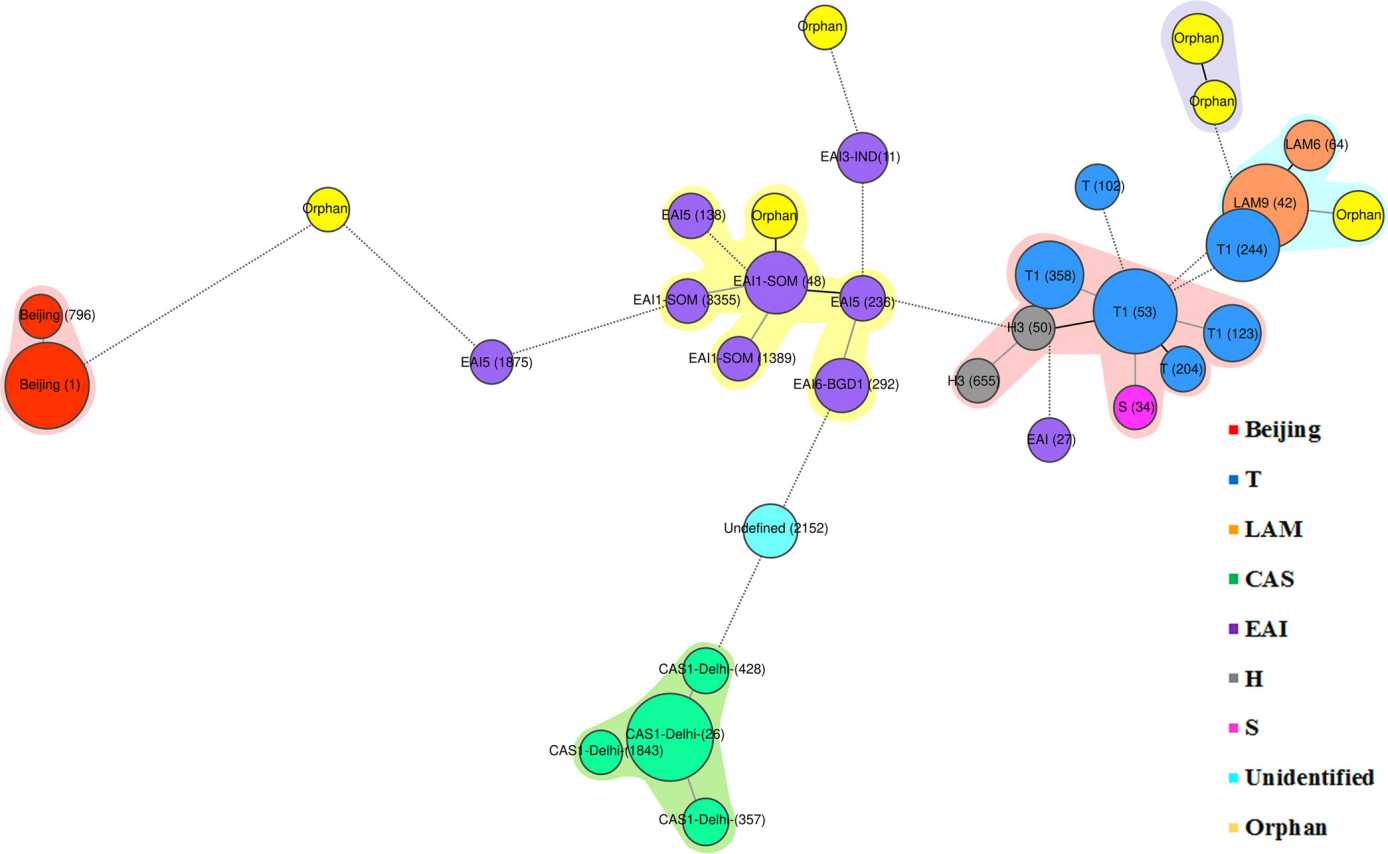
Minimum spanning tree (MST) illustrating the evolutionary relationships between 33 clusters of MDR M. tuberculosis spoligotype lineages. The circles represent clusters of M. tuberculosis isolates and are labeled with their specific spoligotype international type (SIT) numbers. The sizes of the circles are proportionate to the numbers of isolates in each cluster. The color code indicates the major lineages.

Analysis of the spoligotypes revealed that a total of 460 (84.6%) isolates were grouped into 33 distinct clusters with cluster sizes of 2 to 203 isolates. The remaining 84 (15.4%) isolates were unique, with distinct types. The largest cluster comprised 203 (37.3%) isolates of the Beijing genotype (SIT1). Among the other predominant clusters, one comprised 43 (7.9%) isolates of LAM9 (SIT42), while another comprised 36 (6.6%) isolates of CAS1-Delhi (SIT26). There were four clusters under the T1 sublineage, of which the largest comprised 38 (7%) isolates (SIT53), followed by clusters of 19 (3.5%) isolates (SIT244), 17 (3.1%) isolates (SIT358), and 10 (1.8%) isolates (SIT123) (Fig. 1; Table S1). There were a total of nine clusters among EAI lineages, of which the largest cluster was EAI1-SOM (SIT48), containing 14 (2.6%) isolates. Among orphan isolates, there were six clusters ranging from two to four isolates in size. A total of 402 modern type isolates were clustered, yielding a significantly (*P *< 0.001) higher frequency of clustering (402/440; 91.3%) than the ancestral type (58/104; 55.7%).

To visualize and demonstrate the genetic linkages among clusters, we constructed a minimum spanning tree (MST) based on spoligotype data using the MIRU-VNTR*plus* online database (Fig. 1). From the MST, it was evident that most clusters of the same genotypes were close to each other and could be grouped as clonal complexes (CC). It was also apparent that, unlike other lineages, EAI clusters were scattered in the MST. One puzzling result was that an EAI (SIT27) and an S (SIT34) cluster were found to be phylogenetically close to T lineage strains. Moreover, a small number of orphan clusters were also grouped with some CC of defined lineages. This indicated that SIT27, SIT34, or the orphan clusters could be genetically close to those defined lineages, indicating that they are evolutionarily linked.

### Drug susceptibility patterns of different genotypes of MDR M. tuberculosis isolates.

In addition to the two first-line anti-TB drugs (streptomycin [STR] and ethambutol [EMB]), we wanted to investigate the drug susceptibility patterns of MDR-TB isolates for second-line drugs (e.g., kanamycin [KAN], ofloxacin [OFL], and ethionamide [ETH]). Overall, around 81% and 75% of MDR-TB isolates showed resistance to STR and EMB, respectively (Table 3). In terms of second-line anti-TB drugs, approximately 45% of isolates were resistant to ETH and 22% to OFL. Fewer than 1% of isolates (*n* = 5) were resistant to KAN. The frequencies of resistance to STR (*P* < 0.005), EMB (*P* = 0.101), and OFL (*P* < 0.05) were approximately 10% higher for modern-type isolates than for their counterpart, the ancestral type. All KAN-resistant isolates were of the modern type.

**TABLE 3:** Drug resistance patterns of different lineages of MDR M. tuberculosis isolates^*a*^

Classification (no. of isolates)	No. (%) of isolates (*n* = 544) resistant to^*b*^:				
	STR [*n* = 442 (81.2)]	EMB [*n* = 406 (74.6)]	KAN [*n* = 5 (0.9)]	OFL [*n* = 120 (22.0)]	ETH [*n* = 246 (45.2)]
Spoligotype family					
Beijing (208)	177 (85.1)	177 (85.1)	3 (1.4)	41 (19.7)	83 (39.9)
T (93)	86 (92.5)	65 (69.9)	0	16 (17.2)	49 (52.7)
EAI (58)	43 (74.1)	37 (63.8)	0	7 (12.1)	28 (48.3)
LAM (52)	44 (84.6)	43 (82.7)	0	26 (50)	18 (34.6)
CAS (49)	27 (55.1)	27 (55.1)	0	6 (12.2)	21 (42.9)
H3 (5)	3 (60)	2 (40)	1 (20)	1 (20)	2 (40)
S (2)	2 (100)	1 (50)	0	0	0
AFRI-1 (1)	1 (100)	1 (100)	0	0	0
Manu (1)	1 (100)	1 (100)	0	1 (100)	0
SIT2152/1089 (9)	7 (77.7)	5 (55.5)	0	2 (22.2)	5 (55.5)
Orphan (66)	51 (77.3)	47 (71.2)	1 (1.5)	20 (30.3)	40 (60.6)

TbD1 type					
Modern (440)	369 (83.9)	335 (76.1)	5 (1.1)	105 (23.9)	192 (43.6)
Ancestral (104)	73 (70.2)	71 (68.3)	0	15 (14.4)	54 (51.9)

a MDR, multidrug resistant.

b STR, streptomycin; EMB, ethambutol; KAN, kanamycin; OFL, ofloxacin; ETH, ethionamide.

Different lineages exhibited different frequencies of resistance to these five anti-TB drugs. For example, about 93% of T lineage isolates were resistant to STR, while this figure was approximately 85% for the Beijing and LAM lineages (Table 3). Similarly, more than 82.5% of Beijing and LAM lineage isolates were resistant to EMB, while the T lineage was about 70%. Almost 50% of T and EAI lineage isolates and 60.6% of “orphan” isolates were resistant to ETH. The LAM lineage had the highest frequency of OFL resistance, which at 50% was more than twice the overall frequency (22%), and was followed by “Orphan” (28.8%). Analysis of the drug susceptibility profiles of STR, KAN, OFL, and ETH revealed no differences between Beijing and non-Beijing genotypes, except for the EMB resistance, which was found to be significantly higher among Beijing genotypes (*P *= 0.001).

### Clustering analysis of MDR M. tuberculosis isolates.

**FIG 2:**
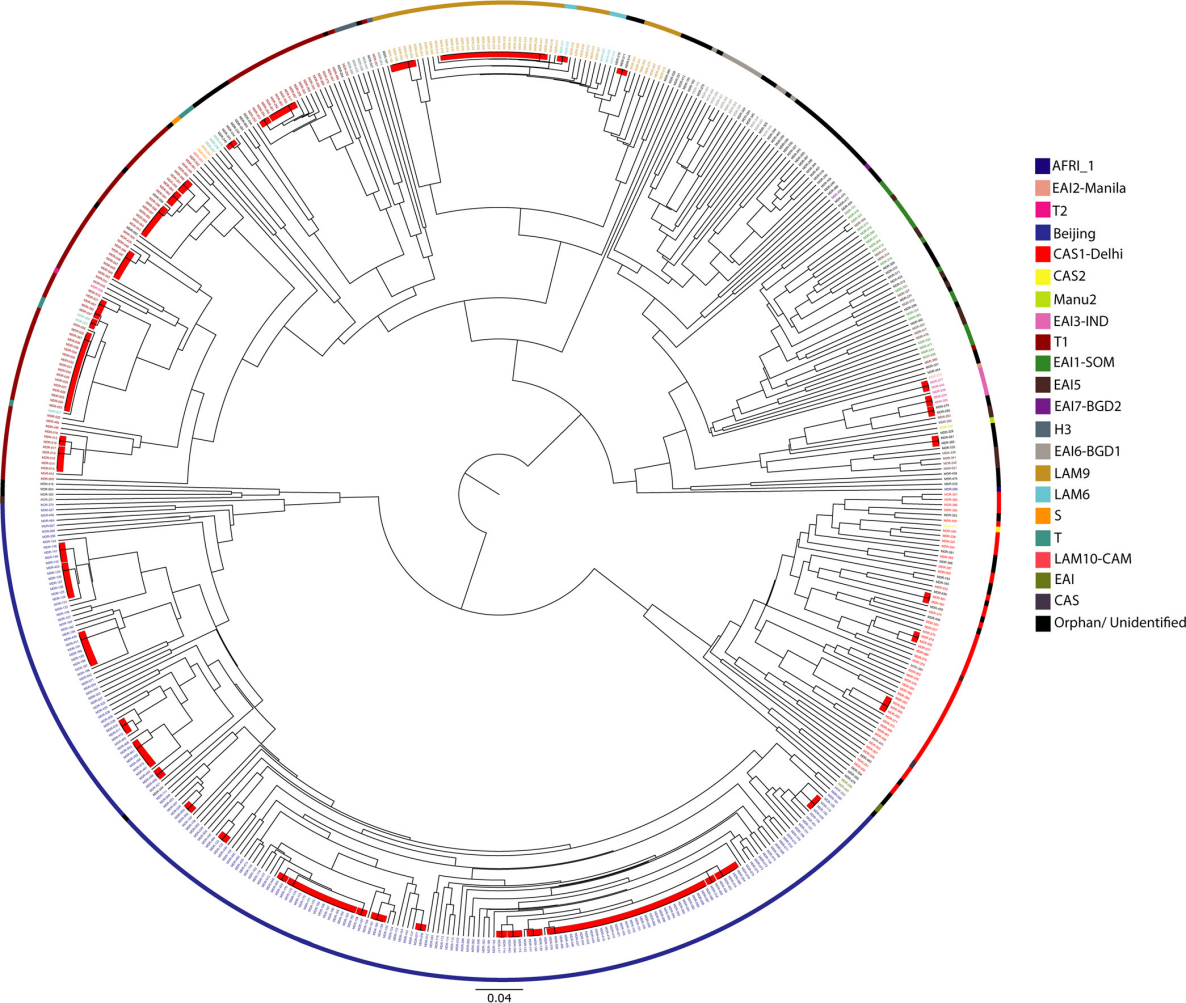
Cladogram showing the phylogenetic relationships among 544 MDR M. tuberculosis isolates as demonstrated by MIRU-VNTR and spoligotyping. The MIRU-VNTR plus web application was used to analyze the data, and FigTree software (http://tree.bio.ed.ac.uk/software/) was applied to construct the cladogram. The outer circular line with different colors denotes different spoligotype lineages, and the inner red bars indicate the clusters as determined by MIRU-VNTR and spoligotyping.

MIRU-VNTR typing of 544 isolates using 15 loci resulted in 341 different patterns with 49 clusters containing 252 isolates (cluster size ranging from 2 to 34 isolates). Moreover, 24-locus MIRU-VNTR resulted in 360 different patterns with 43 clusters containing 227 isolates (cluster sizes ranging from 2 to 34 isolates). The clustering rates by individual spoligotyping, 15-locus, and 24-locus MIRU-VNTR typing were 84.6%, 46.3%, and 41.7%, respectively. However, the combination of spoligotyping and 24-locus MIRU-VNTR yielded 378 different patterns with 44 clusters containing 209 isolates, and the clustering rate was reduced to 38.4% (Table 4). The phylogenetic relationships among the studied strains, as demonstrated by the combination of MIRU-VNTR and spoligotyping, are shown in Fig. 2. The 208 isolates of Beijing genotypes were further grouped into 120 distinct patterns by MIRU-VNTR typing, which consisted of 20 clusters containing 108 isolates; the remaining 100 isolates had unique patterns. The largest cluster contained 33 isolates of the Beijing genotype (SIT1). Among other major sublineages, T1 (*n* = 86) exhibited 11 clusters containing 54 isolates, of which the largest had 16 isolates, and LAM9 (*n* = 46) exhibited two clusters containing 26 isolates, of which the largest had 21 isolates (Fig. 2). The remaining clusters ranged from two to eight isolates in size. The rate of clustering among Beijing strains (51.9%; 108/208) was significantly higher than among non-Beijing strains (30.7%; 103/336) (*P* < 0.001). The clustering data also demonstrated that 31 (70.1%) of 44 clusters were formed by both Beijing and T1 lineages, which contained 77.5% (162/209) of the total clustered isolates. Beijing made up 51.7% (108/209) of the total clustered isolates, followed by T1 at 25.8% (54/209). In this analysis, we also found that the clustering rate was higher among modern MDR-TB strains (49.3%; 217/440) than among ancestral strains (9.6%; 10/104) (*P* < 0.001).

**TABLE 4:** Clustering of M. tuberculosis isolates using spoligotyping, MIRU-VNTR, and the combination of both methods^*a*^

Classification method^*b*^	No. of:			Cluster size (no. of isolates)	No. of unique isolates	Clustering rate (%)
	Patterns	Clusters	Clustered isolates			
Spoligotyping	117	33	460	2–203	84	84.6
MIRU-VNTR using						
15 loci	341	49	252	2–34	292	46.3
24 loci	360	43	227	2–34	317	41.7
Spoligotyping + 24-locus MIRU-VNTR	378	44	209	2–33	335	38.4

a *n* = 544 isolates.

b MIRU-VNTR, mycobacterial interspersed repetitive-unit–variable-number tandem-repeats.

### Geographic distribution and transmission patterns of MDR-TB strains.

**FIG 3:**
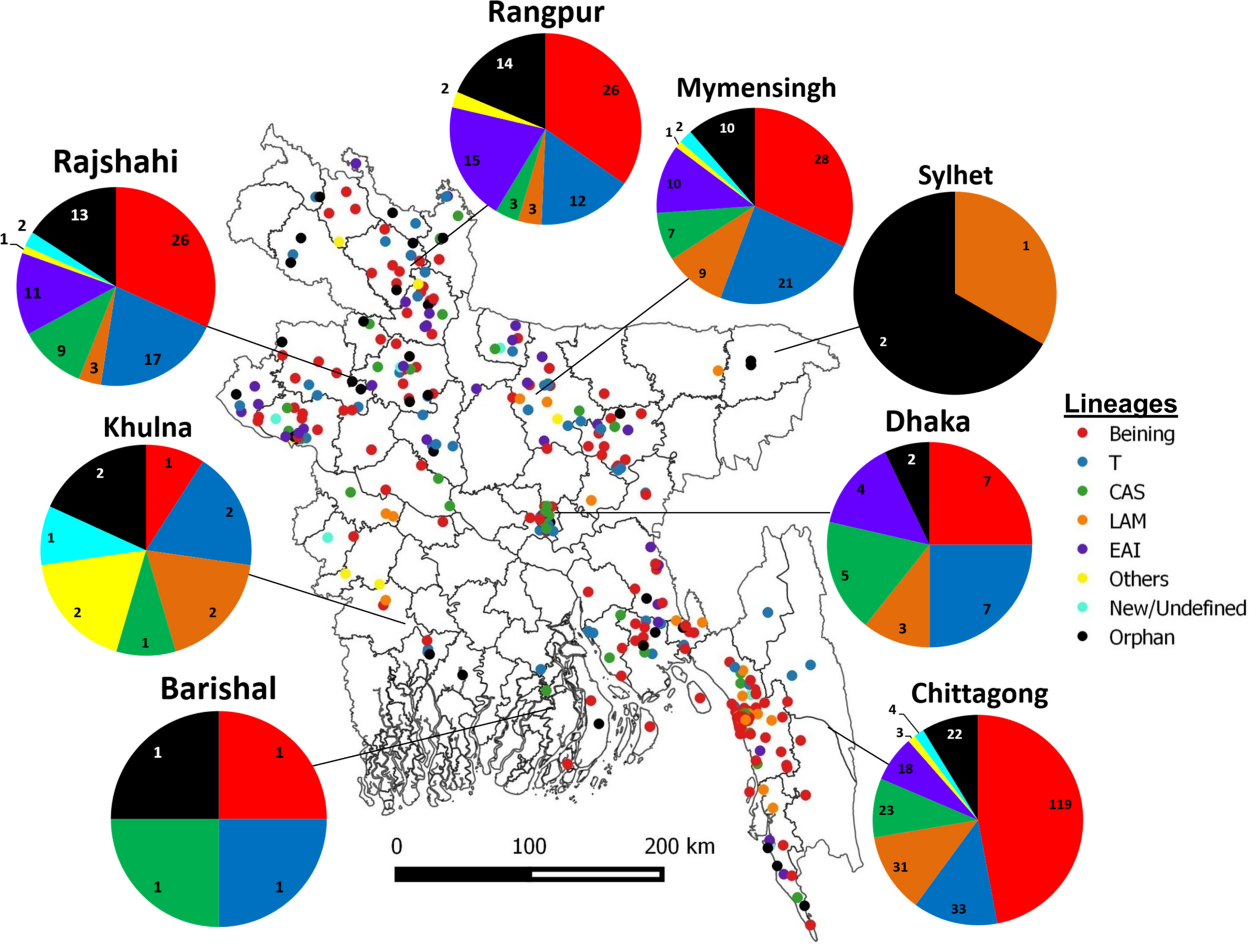
Distribution of different spoligotype lineages of 544 MDR M. tuberculosis isolates in different geographical region of Bangladesh. The distribution of lineages in each geographical region is shown in the map, as well as in the corresponding pie-chart. The map was generated using the software ‘QGIS2.10.1-Pisa’.

In the current study, most of the MDR-TB isolates were obtained from the Chittagong, Mymensingh, Rajshahi, and Rangpur divisions. The distribution of different lineages is shown in Fig. 3. Almost half of the isolates from the Chittagong division (47%; 119/253) were of the Beijing lineage, which was higher than the overall Beijing frequency of this analysis; this was followed by Rangpur (34.7%; 26/75), Mymensingh (31.8%; 28/88), and Rajshahi (31.7%; 26/82). The T lineage was the second most predominant; T strains were found in all divisions, but their numbers were higher in Mymensingh (23.9%; 21/88), Dhaka (25%; 7/28), Rajshahi (20.7%; 17/82), and Rangpur (16%; 12/75). An abundance of LAM and CAS strains was found in the Chittagong, Mymensingh, Dhaka, and Rajshahi divisions, whereas the frequencies of EAI lineage strains were higher in the Rangpur (20%; 15/75) and Rajshahi (13.4%; 11/82) divisions. There were some minor sublineages found in this analysis, such as H3, S, AFRI-1, and MANU2, which contributed less than 10% of isolates in each division but were not exclusive to any specific location (Fig. 3).

**FIG 4:**
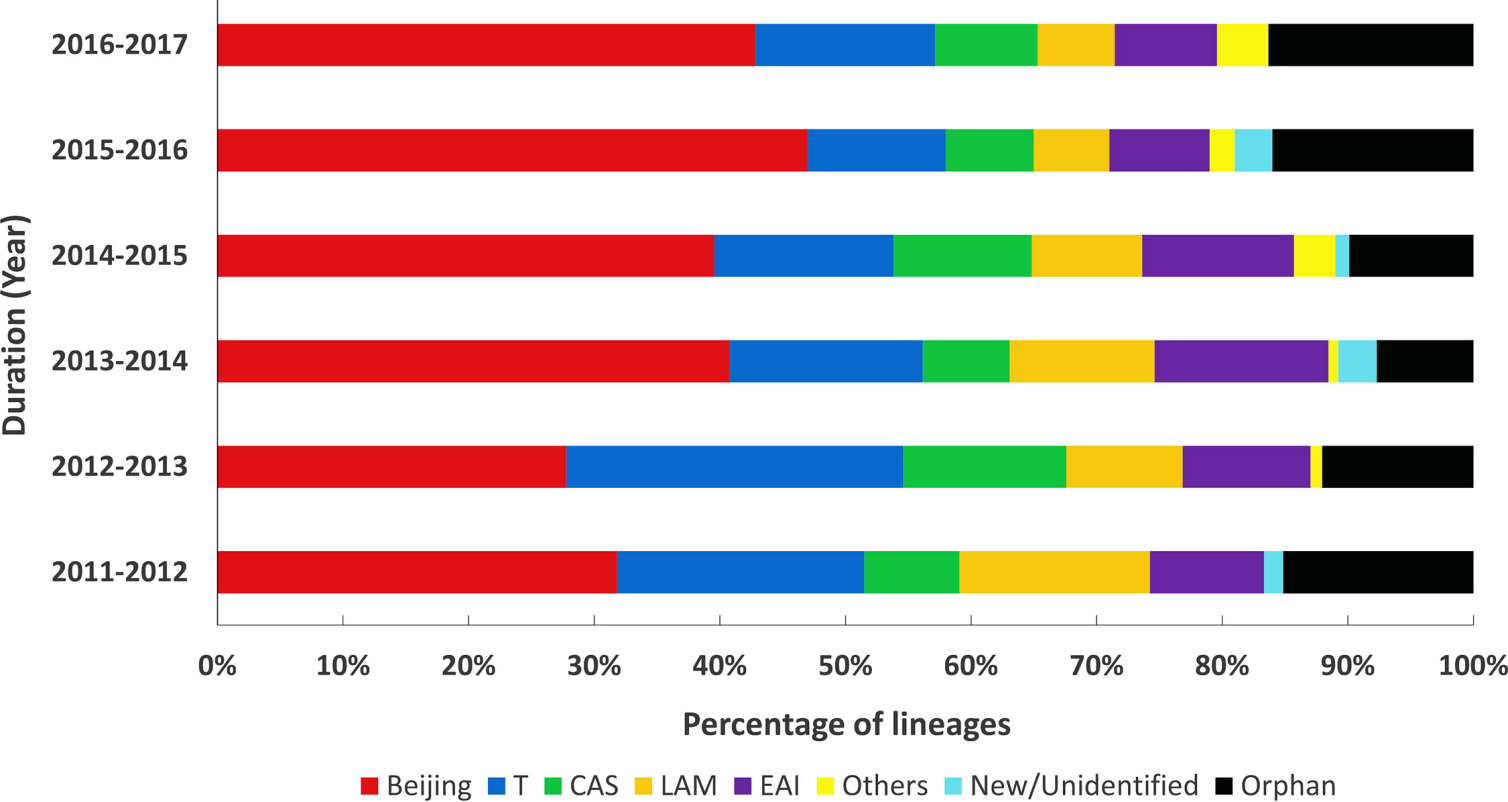
Distributions of MDR-TB spoligotype lineages in different time frames throughout the study period. Compared to other lineages, Beijing was the most prevalent throughout the study period and demonstrated a rising trend from around 30% in 2011 to 2013 to nearly 50% in 2015 to 2017.

We also analyzed the year-wise distribution of MDR-TB spoligotype lineages (Fig. 4). The Beijing genotype was the most prevalent throughout the study period, exhibiting a rising trend from around 30% in 2011 to 2012 to nearly 50% in 2015 to 2017. MDR isolates of the T lineage were the next most predominant genotype found early in the study period but decreased considerably over time. Isolates belonging to the CAS and EAI lineages were found at nearly the same rates each year, although LAM isolates tended to decrease across the study period. There was no significant difference in the distribution of other lineages, unidentified strains, or orphan strains.

**FIG 5:**
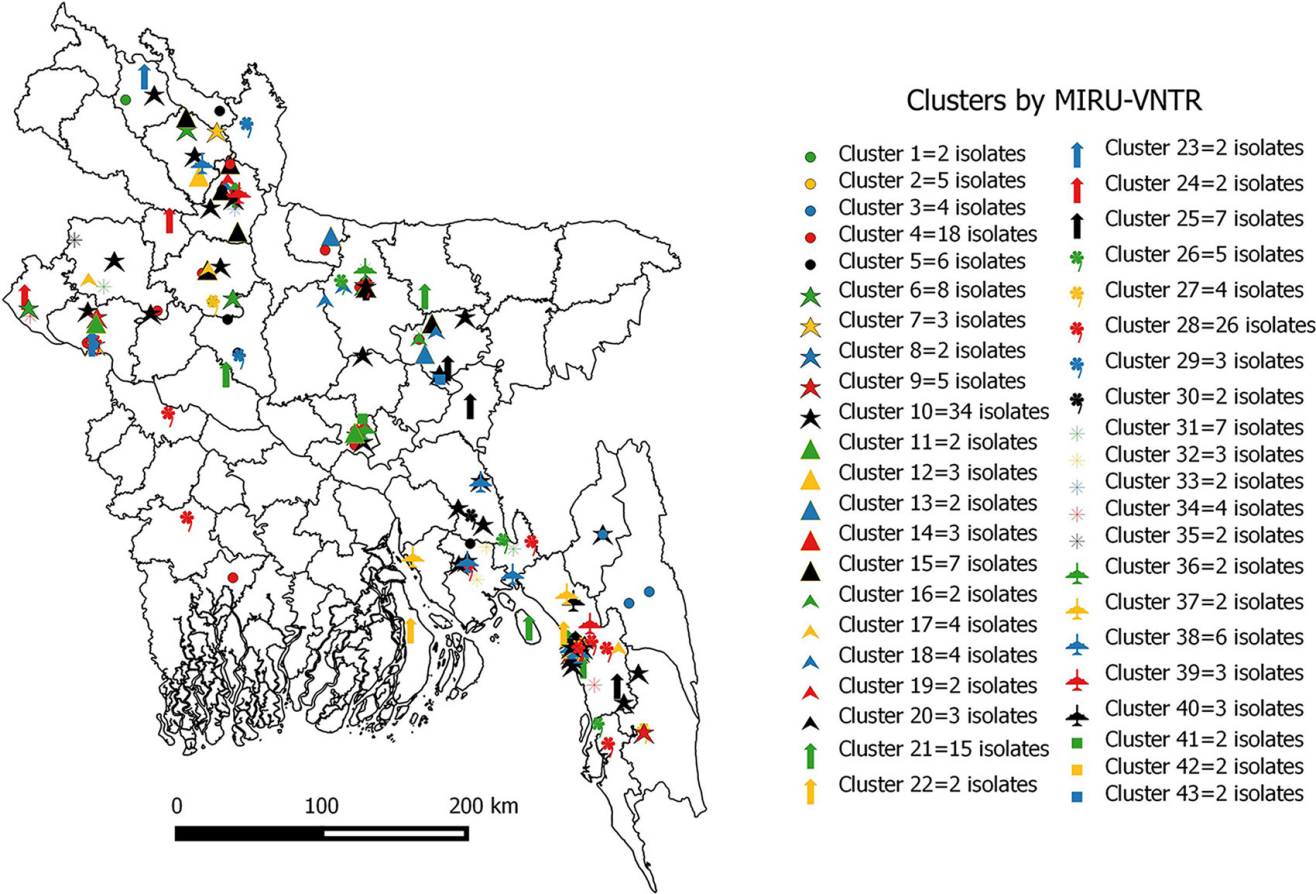
Map of Bangladesh showing the distribution of clustered MDR M. tuberculosis isolates as determined by the MIRU-VNTR typing method. Each color and shape denotes a different cluster. The map was generated using the software ‘QGIS2.10.1-Pisa’.

The transmission pattern of MDR-TB isolates among clustered strains was analyzed over the study period. The rate of recent transmission over the entire study period was 33.8%, where *T_c_* = 227, *N_c_* = 43, and *T_a_* = 544 (*T_c_* represents the total number of clustered strains, *N_c_* is the number of clusters, and *T_a_* is the total number of isolates). The rates of both clustering and recent transmission were higher in the first and second 22-month periods of the study than in the third 22-month period (Table 5). The rates of recent transmission were 28.6% and 28.8% in the first and second 22 months of the study, respectively, compared to 21.4% in the third 22-month period. The overall distribution of 227 clustered isolates in different geographical areas of Bangladesh is illustrated in Fig. 5. The clustering rate was found to be higher in the Chittagong (42.3%) and Mymensingh (42%) divisions than in Rajshahi (28%), Dhaka (21.4%), and Rangpur (20%) (*P* < 0.05). There was only one cluster of two isolates in Khulna division, and all isolates from the Barishal and Sylhet divisions were unique.

**TABLE 5:** Clustering of M. tuberculosis isolates during different time intervals analyzed using MIRU-VNTR typing

Time period (no. of isolates obtained)	Duration	No. of:		Cluster size (no. of isolates)	Rate (%) of:	
		Clusters	Clustered isolates		Clustering	Recent transmission
Overall (544)	October 2011 to March 2017	43	227	2–34	41.7	33.8
1st (168)	October 2011 to July 2013	19	67	2–10	39.9	28.6
2nd (222)	August 2013 to May 2015	20	84	2–14	37.8	28.8
3rd (154)	June 2015 to March 2017	15	48	2–14	31.2	21.4

## DISCUSSION

Although Bangladesh is a country with high TB and MDR-TB burdens, there has been no systematic analysis of the association of strain genotypes with the drug resistance and transmission dynamics of MDR-TB. To our knowledge, this is the first in-depth analysis of genetic diversity and transmission dynamics of MDR-TB isolates collected over a multiyear period and covering all geographical divisions in Bangladesh.

In this analysis, we genotyped 544 MDR-TB isolates collected over a 6-year period using TB-specific deletion (TbD1) analysis and spoligotyping. TbD1 analysis revealed that around 81% of MDR-TB cases were due to infection caused by modern-type M. tuberculosis strains. This finding is unsurprising, as previous studies have reported modern isolates to be associated with increased drug resistance and MDR-TB (15, 16). Brosch et al. have suggested that the TbD1 deletion may confer some selective advantages to modern M. tuberculosis strains (14). Moreover, modern genotypes have increased virulence or transmissibility, which may have evolved as a response to coevolutionary interactions with particular human populations, mass Mycobacterium bovis BCG (BCG) vaccination, and/or the use of anti-TB drugs (17). Earlier studies from our group that had a considerable number of MDR-TB isolates found a high frequency (approximately 70%) of modern-type strains (18). Our neighboring country, Myanmar, has also reported a high prevalence of modern Beijing genotype among MDR-TB cases (19). In contrast, our previous studies with non-MDR patients in rural Matlab and the Northeast part of Bangladesh found a higher prevalence (>65%) of ancestral lineages (7, 20).

Through spoligotyping, we were able to determine the genotype of about 88% of isolates in this analysis; the remainder were new or unknown genotypes. We observed widely diverse spoligotype patterns (*n* = 127), reflecting the polymorphisms among the MDR-TB strains. The main genotype responsible for the MDR-TB in our study was Beijing, which accounted for 38.2% of total isolates. The frequent association of Beijing genotype with MDR-TB has been reported in many Asian countries (4, 5, 21–26) and one of our previous studies (18). The prevalence of Beijing genotypes among MDR-TB patients in our study is consistent with results from the neighboring country of India (29 to 35%) (21, 22) but is lower than in other Asian countries, such as Nepal (48.4%) (23), Vietnam (71%) (24), Thailand (72.4%) (4), Myanmar (79.2%) (19), and China (75 to 94%) (5, 25, 26). The exact reason for the association of the Beijing genotype with drug resistance remains unresolved. However, some researchers have hypothesized that a higher mutation rate in the genome of the Beijing genotype could confer accelerated acquisition of drug resistance or could efficiently compensate for the negative effect of the anti-TB drugs (27).

In the current study, the occurrence of the Beijing genotype was found to be higher in the Chittagong, Rajshahi, Rangpur, and Mymensingh divisions. However, this finding was not reflected in other geographical divisions, including Dhaka and Barishal, despite these two divisions sharing a border with Chittagong. This might be due to the fact that the MDR-TB strains obtained from the surveillance were not homogenously distributed across all divisions, making it difficult to determine the precise distribution of the Beijing genotype. Approximately 92% of MDR-TB isolates were obtained from the divisions of Chittagong, Rajshahi, Rangpur, and Mymensingh; this is because these divisions are home to three dedicated chest disease hospitals for the treatment of MDR-TB patients, and as sentinel surveillance sites, most suspected or confirmed MDR-TB patients admitted to these facilities were enrolled for XDR-TB surveillance. Future studies should be conducted to determine whether this higher proportion of the Beijing genotype is spread consistently across all geographical divisions.

Diverse non-Beijing genotypes also represented almost half of the studied strains. Non-Beijing strains mainly included T (17.1%), EAI (10.7%), LAM (9.5%), and CAS (9.1%). In this study, about 17% of MDR-TB isolates were T type, making it the second most prevalent lineage after Beijing. This finding is consistent with our earlier published reports, which warned that the emergence of drug-resistant TB in Bangladesh could be attributed largely to Beijing and T lineage strains (18). EAI and CAS are mostly geographically specific to Southeast Asia, the Indian subcontinent, Western Asia, and East Africa (28, 29). A recent meta-analysis has revealed a significantly higher proportion of MDR-TB strains among the CAS lineage than for EAI (29). EAI and CAS lineages among MDR-TB strains were also reported by our previous studies (18, 20), as well as by neighboring countries, such as India (30), Nepal (23), and Myanmar (19). Studies from Latin American countries have reported the LAM lineage, which is strongly associated with drug resistance and MDR-TB (31, 32). However, a report from Brazil has shown a high proportion of T and H lineages among MDR-TB isolates (13). The most important concern is that, in addition to some lineages that are geographically specific to Bangladesh (e.g., Beijing or EAI), diverse clones of virulent strains (e.g., LAM, T, H, and S) are also circulating and contributing to MDR-TB. However, we could not find any homogenous distribution of MDR strain genotypes across geographical divisions, which is unsurprising. Furthermore, the distribution of MDR-TB strains across different geographical divisions does not correlate precisely with the overall prevalences of TB in those divisions. This is due to the fact that the MDR-TB isolates used in this study for molecular characterization were obtained from a sentinel surveillance program aimed at detection of MDR- and XDR-TB in Bangladesh. The primary objective of this surveillance was to determine the rates of resistance in the country, along with their risk factors, rather than to assess geographical differences. Hence, the sample size did not have sufficient power to establish geographical variance. The distribution discussed in the study is descriptive; further research is recommended to address the association of the geographic differences in MDR-TB strains with the overall TB prevalences.

The overall clustering rate (41.7%) identified by MIRU-VNTR in our study is comparable with those of other, earlier reports, in which MDR-TB exhibited higher clustering rates (34 to 37%) than non-MDR or monoresistant isolates (33, 34). Clustering rates can vary depending on study design and setting or any link to an outbreak, which complicates any comparison of clustering and transmission rate consistency across studies. A study from China showed 18% clustering with 8.7% transmission for MDR-TB (35); in contrast, a study from Myanmar had a 48.6% clustering rate (19). It has been well established that Beijing lineage strains are highly transmissible. Our data demonstrated that the rate of clustering of Beijing strains (51.9%) was significantly higher than that of non-Beijing strains (30.7%). Several earlier studies also reported higher clustering for Beijing strains (16.3 to 45.2%) than for non-Beijing types (2.7 to 28.6%) (5, 25). Combined with the abundance of Beijing-type strains, their higher clustering rates and a rising trend across the entire study period imply that this lineage, compared to others, may lead to a future outbreak of MDR-TB in Bangladesh.

Upon closer inspection of the genetic clusters, many of them lacked any obvious epidemiological linkage. Thus, if we define a cluster as isolates having 100% similarity in epidemiological and genetic data, we will miss many isolates from a cluster, which might lead to misinterpretation of the actual transmission. It is therefore also important to note that the identification of transmission clusters does not necessarily imply that the actual transmission occurred at the place and time studied. It may reflect transmission that occurred earlier and the subsequent progression of active disease. Accordingly, we examined different epidemiological linkage data and the drug resistance phenotypes of clusters and found that many isolates from the same clusters were collected at a different time in the study, as well as from different geographical locations. This suggests that, unless direct epidemiological linkage is found, clustered genotypes should be interpreted with caution for recent transmission of MDR-TB. Similar phenomena have also been found in some earlier studies, with similar advice given regarding transmission analysis (32).

Moreover, we investigated the associations between drug susceptibility patterns and the genotypes of MDR-TB isolates. Most modern lineages, such as Beijing, T, and LAM, had high frequencies of STR and EMB resistance (∼70 to 92%). Although EMB resistance was found to be significantly higher for the Beijing genotype, this was distributed throughout the clade and not in any particular cluster. In contrast, 55 to 75% of EAI, CAS, and orphan isolates were resistant to these two first-line drugs. In terms of second-line drugs, such as OFL and KAN, strains of most modern lineages (e.g., Beijing, H, LAM, and T) showed more resistance than EAI and CAS. Additionally, about 1% of isolates (*n* = 5) in this study were resistant to KAN and fluoroquinolones, meaning that they are XDR-TB. These five isolates were all modern type (lacking the TbD1 region [TbD1^−^]), with three of them being Beijing, one being H3, and the remaining one being Orphan type. Altogether, these findings support the previously discussed phenomenon of the comparative disposition of resistance phenotypes among modern lineages relative to their ancestral counterparts (36). In the current investigation, we focused only on MDR-TB isolates; as a result, we could not compare the MDR-TB strain distribution to that of drug-sensitive TB patients. In a recent study, we found that the EAI lineage was the most prevalent (at around 68%) among drug-sensitive TB patients in the Northeast part of Bangladesh, whereas Beijing was the most prevalent (at around 42.0%) among MDR-TB patients (20). Future research should be carried out across the country to investigate the strain differences between MDR-TB and drug-sensitive TB patients.

Direct contact with TB patients or risk group population is an important component of evidence for transmission. According to 2010 WHO drug resistance surveillance data, many MDR-TB cases occur among previously untreated patients, which rules out the possibility of endogenous/acquired resistance and is an indication of a serious result from the transmission (37). In this study, 451 participants had no history of exposure to any TB patients, which rules out the possibility of direct transmission to around 83% of the study population. However, 501 patients in this study had a history of TB. Additionally, we were able to collect the treatment outcome data of previous TB infection episodes for 413 of 500 treated patients; these findings revealed that about 58% of patients had experienced treatment regimen failure or did not complete the treatment. This high frequency of MDR among participants with no contact history but who had been previously treated with anti-TB drugs reflects development through selective pressure, in which a small population of bacteria genetically mutate to provide drug resistance. This phenomenon can also indicate that the patients with current infection episodes (92% of patients in this analysis) might have experienced a recurrence of their earlier infections. As we do not have the susceptibility data from earlier infections (paired samples from the same patient), we were unable to demonstrate sensitive-to-resistant conversion, which would enable us to confidently draw conclusions regarding the real acquired resistance. A study from China with paired samples (two different episodes of infection) from the same patients showed that about 41% of drug resistance among previously treated patients was endogenously acquired (38). An earlier study by our group in a tertiary referral hospital described the high frequency of MDR among previously treated patients (18). In contrast, the rate of about 60% of isolates without clustering in the current study indicated that a majority of MDR cases were acquired. It is worth mentioning that Bangladesh began the implementation of the GeneXpert assay in 2012; before that, most primary TB patients were treated based solely on the findings of sputum microscopy, without awareness of the drug resistance status of the TB isolates. For this reason, many RIF-resistant patients may have remained undetected and received anti-TB treatment with first-line drugs. There is a chance that these patients later developed MDR-TB due to the use of inappropriate treatment regimens (39). Future studies should be performed to distinguish between acquired and primary resistance among MDR-TB patients all over the country, as this would aid in the formulation of a strategy for better control of MDR-TB. If the majority of cases of drug resistance emerge as a result of acquired resistance, emphasis must be placed on strengthening the effective treatment, as well as tackling the factors related to regimen failure. If, however, primary resistance is a major factor, the creation and implementation of transmission control programs must be added as a critical component of the control strategy (38).

These MDR-TB samples were collected in a span of around 6 years, from October 2011 to March 2017. We divided this period into 22-month slots and calculated clustering and recent transmission rates for each. It was evident that both clustering and recent transmission rates decreased gradually in every slot, from a 39.9% clustering rate in the first 22-month period to a rate of 31.2% in the final 22 months. Consequently, the rate of recent transmission also decreased by more than 6%. This gradual decrease may be a reflection of the strengthening of nationwide vigorous screening of MDR-TB patients and successful treatment.

The limitation of current molecular typing lies in the utilization of culture-based DNA, which takes about 4 to 6 weeks due to the slow-growing nature of TB bacilli; by the time molecular epidemiological data have been generated, it is already too late to begin the intervention. This confines the use of molecular epidemiological tools to retrospective studies rather than real-time infection control (40). Although there are enormous associated challenges, whole-genome sequencing (WGS), possibly directly from specimens, could be an attractive tool for earlier and real-time genotyping, drug resistance determination, and better understanding and delineation of transmission clusters (40, 41). We further acknowledge the limitation that the MDR-TB patients included in this analysis were obtained from selected hospitals by following a systematic random sampling strategy of sentinel surveillance for detection of MDR- and XDR-TB rather than from a focused study on transmission dynamics (42). Therefore, we were not able to enroll all MDR-TB patients who visited the hospitals. As a result, despite the occurrence of significant transmission, we may have missed the actual epidemiological links among the MDR-TB patients (if any); hence, the current analysis might not reflect the true scenario of transmission at the population level. Moreover, as this surveillance sampling was based on the entire country’s prevalence of MDR-TB rather than a particular geographical location, it was statistically challenging to establish the predominance of a particular lineage in a particular region. Further studies should be performed to facilitate a more detailed exploration of the molecular characterization and transmission dynamics of MDR-TB in different geographical divisions by including all MDR-TB patients.

In conclusion, this study provides the first insight into the molecular characterization and transmission dynamics of MDR M. tuberculosis isolates in Bangladesh. Our results demonstrate that the MDR M. tuberculosis isolates in circulation are mostly the modern virulent type. Modern MDR-TB isolates exhibited a higher clustering rate, as well as higher resistance to other anti-TB drugs, than did the ancestral types. Among genetically diverse MDR-TB isolates, the Beijing and T lineages are the predominant types to which most transmission can be attributed. The molecular epidemiological analysis coupled with previous treatment/contact history suggests that, along with the remarkable transmission, the emergence of MDR-TB in Bangladesh may be largely attributed to acquired resistance resulting from nonadherence to treatment or inadequate treatment of TB patients. Finally, the current study findings will provide a better understanding of MDR-TB strain diversity and transmission dynamics in Bangladesh, which will ultimately lead to more effective strategies for the programmatic management of MDR-TB.

## MATERIALS AND METHODS

### Specimens.

The MDR M. tuberculosis isolates were obtained from a nationwide sentinel surveillance study aimed at detection of MDR- and XDR-TB that was conducted by the International Center for Diarrheal Diseases Research, Bangladesh (icddr,b) between October 2011 and March 2017, which included 23 hospitals covering all geographic divisions of Bangladesh (42). For the surveillance of MDR-TB, samples were collected from newly registered smear-positive pulmonary TB patients, which included both new TB cases and previously treated TB cases (relapse, failure, or lost to follow-up). Participants were sampled following a systematic random sampling strategy with a selection of every 2nd/5th/10th eligible patient, depending on the flow of patients in the individual health care facility (42). For the surveillance of XDR-TB, all suspected or known MDR-TB patients admitted to the health care facilities were enrolled. The sample size for this surveillance testing was calculated by considering the overall 7% prevalence of MDR-TB in Bangladesh. We required 625 samples to estimate this level of prevalence with a 2% precision and 5% alpha level. Considering an estimated loss of 20%, we finally arrived at a sample size of 750 TB patients to be screened per year to achieve a total of 4,125 patients for the overall surveillance period of 5.5 years. We eventually included 544 MDR-TB isolates in the current investigation, based on the available results for culture growth, drug susceptibility testing, and all molecular typing methods. Patients were excluded from participation in the study if they were already undergoing treatment with an anti-TB drug at the time of diagnosis as a smear-positive case. The study was approved by the Research Review Committee (RRC) and Ethical Review Committee (ERC) of icddr,b. Participants were included in the study only after their written informed consent was obtained.

### Culture and drug susceptibility testing (DST).

Sputum specimens were processed and decontaminated using a previously described method (43). The decontaminated specimens were inoculated onto two solid Lowenstein-Jensen (LJ) medium slants and incubated at 37°C. If no visible growth was observed on either of the LJ medium slants within 8 weeks, the specimen was considered culture negative. The drug susceptibilities of M. tuberculosis isolates to isoniazid (INH; 0.2 μg/μL), rifampicin (RIF; 40 μg/μL), ethambutol (EMB; 2 μg/μL), streptomycin (STR; 4 μg/μL), ethionamide (ETH; 40 μg/μL), kanamycin (KAN; 30 μg/μL), and ofloxacin (OFL; 2 μg/μL) were tested using the standard LJ proportion method described in previous works (43, 44). If any growth of 1% or more compared to the growth on the control (drug-free) medium was observed in any drug-containing medium, the isolate was considered resistant to the given drug. Sputum sample processing, culture, and drug susceptibility testing of the cultured MDR-TB isolates were performed in biosafety level 2 (BSL-2)-plus and BSL-3 facilities at icddr,b.

### DNA extraction and deletion analysis.

Genomic DNA was extracted using the cultured mycobacterial colonies as previously described (6). The presence or absence of M. tuberculosis*-*specific deletions was determined as described previously by using PCR with internal and flanking primers (14). Isolates were confirmed as M. tuberculosis based on the presence of the RD9 region, as this is strictly conserved in M. tuberculosis. Isolates having the TbD1 region intact (TbD1^+^) were considered the “ancestral” type, while isolates lacking the TbD1 (TbD1^−^) region were considered the modern type of M. tuberculosis (14).

### Spoligotyping and MIRU-VNTR typing.

Spoligotyping was carried out with genomic DNA of MDR-TB isolates using a commercially available kit (Isogen Biosciences, BV, Bilthoven, Netherlands) and following the standard protocol described in prior research (45). The genomic DNAs of M. tuberculosis strain H37Rv and M. bovis BCG P3 were used as positive controls, while distilled water was employed as a negative control. Spoligotyping results were converted into an octal format and compared with the international spoligotyping database using SITVIT2, an updated version of SITVITWEB (http://www.pasteur-guadeloupe.fr:8081/SITVIT2/index.jsp) (46). MIRU-VNTR typing by PCR was performed initially for all isolates using 15 loci (MIRU4, MIRU10, MIRU16, MIRU26, MIRU31, MIRU40, Mtub04, ETR C, Mtub21, QUb-11b, ETR A, Mtub30, Mtub39, QUB-26, and QUB-415). If any cluster was found using 15 loci, then testing with an additional 9 loci (MIRU2, MIRU20, MIRU23, MIRU24, MIRU27, MIRU39, Mtub29, ETR B, and Mtub34) was conducted to further discriminate among the clustered isolates. PCR amplification was performed on 96-well microplates using a standard protocol (47). The PCR products were then analyzed using the automated QIAxcel advanced system (Qiagen AG, Germany) as described previously (12, 13). The copy numbers of different MIRU-VNTR loci for each strain were entered into the MIRU-VNTR*plus* web application (http://www.miru-vntrplus.org/MIRU/index.faces) to define the clustering patterns. This MIRU-VNTR*plus* online application program was used to construct an unweighted pair group method with arithmetic mean (UPGMA) tree using both MIRU-VNTR and the spoligotyping data of MDR-TB strains (20). To evaluate phylogeny convergence, data obtained from the MIRU-VNTR*plus* tool were imported into FigTree software (version 1.4.4) (http://tree.bio.ed.ac.uk/software/) to construct a cladogram.

### Epidemiological investigation.

A cluster was defined as two or more isolates from different patients having identical spoligotype or MIRU-VNTR patterns, whereas nonclustered patterns were categorized as unique. Clustered MDR-TB patients were investigated to further establish any potential epidemiological connections concerning place, time, and person among cluster members. Participants were considered to share an apparent epidemiological link if they had been in the same workplace, household, village, or area at overlapping times. The rate of recent transmission was calculated using the formula (*T_c_* − *N_c_*)/*T_a_*, where *T_c_* represents the total number of clustered strains, *N_c_* is the number of clusters, and *T_a_* is the total number of isolates (7, 48).

### Data analysis.

Data were entered and analyzed using the SPSS 20.0 software package (Statistical Package for the Social Sciences, Inc., Chicago, IL, USA). Fisher’s exact or the *χ*^2^ test was used to determine the associations between strain types and demographic or clinical information. A *P* value of <0.05 was considered evidence of a significant difference.
