# Supplementary material for: Molecular Epidemiology and Genetic Diversity of Multidrug-Resistant Mycobacterium tuberculosis Isolates in Bangladesh
Source: Microbiol Spectr. 2022 Feb 23;10(1):e01848-21. doi: 10.1128/spectrum.01848-21 (PMC8865560; doi:10.1128/spectrum.01848-21)
Supplement: SUPPLEMENTAL FILE 1 — Supplemental material. Download SPECTRUM01848-21_Supp_1_seq9.pdf, PDF file, 0.2 MB [file spectrum01848-21_supp_1_seq9.pdf]

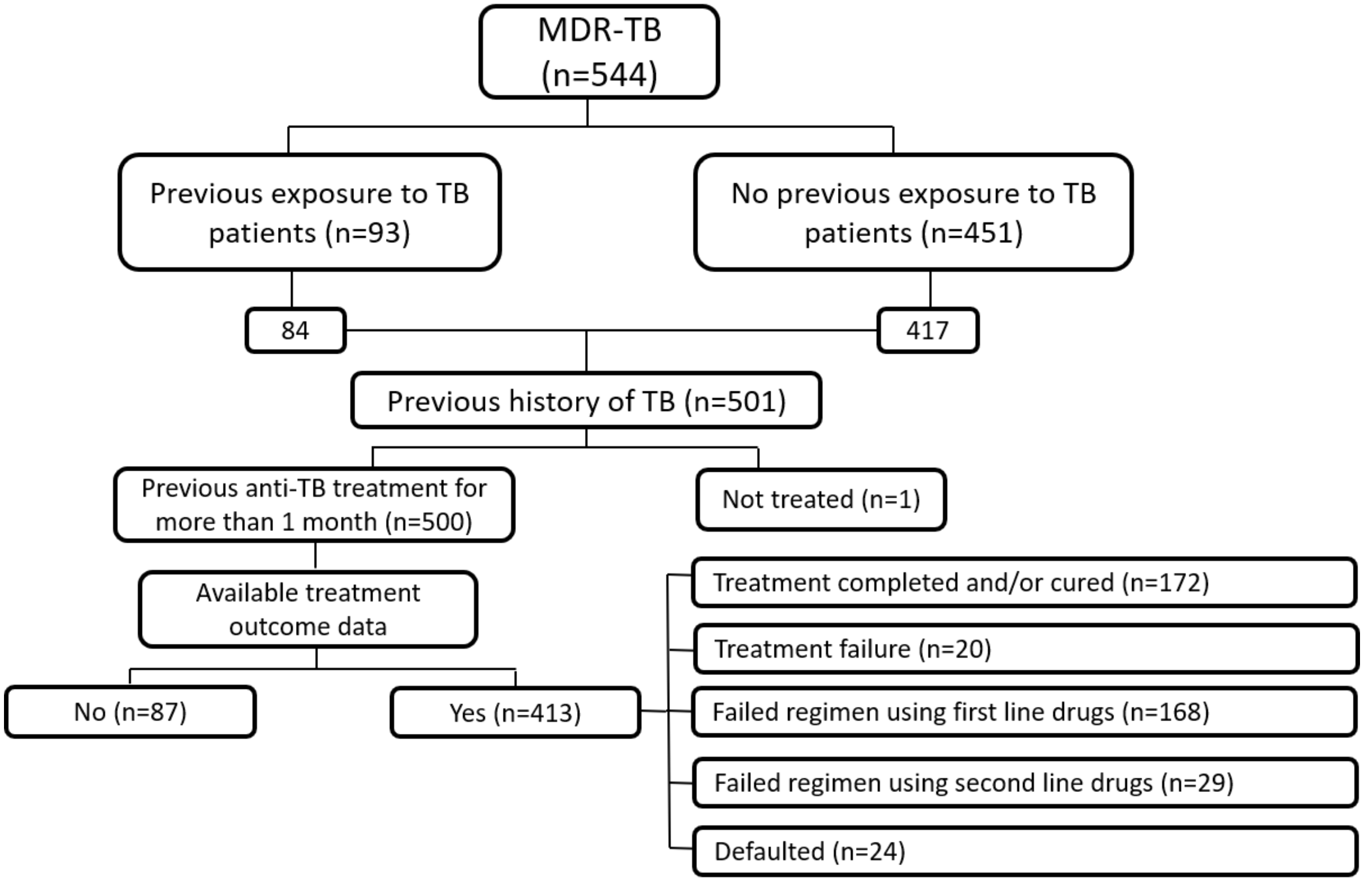

Fig. S1 Flow chart showing the previous exposure and/or tuberculosis history and treatment outcome of 544 MDR tuberculosis patients included in the study

Table S1 Details of *M. tuberculosis* lineages and sub-lineages with their corresponding SIT numbers, octal codes, and spoligotype pattern found among 544 MDR-TB patients in Bangladesh

Lineage (n)	SIT	Sub-lineage	Octal code	Spoligotype pattern	No. of isolates, n (%)
Beijing (208)	1	Classical Beijing	000000000003771	□□□□□□□□□□□□□□□□□□□□□□□□□□□□□□□□□□■■■■■■■■■	203 (37.3)
	265	Non-classical	000000000003371	□□□□□□□□□□□□□□□□□□□□□□□□□□□□□□□□□□■■□■■■■■■	1 (0.2)
	269	Non-classical	000000000000771	□□□□□□□□□□□□□□□□□□□□□□□□□□□□□□□□□□□□■■■■■■■	1 (0.2)
	796	Non-classical	000000000001771	□□□□□□□□□□□□□□□□□□□□□□□□□□□□□□□□□□□■■■■■■■■	2 (0.4)
	2610	Non-classical	000000000003770	□□□□□□□□□□□□□□□□□□□□□□□□□□□□□□□□□□■■■■■■■■□	1 (0.2)
CAS (49)	26	CAS1-Delhi	703777740003771	■■■□□□□■■■■■■■■■■■■■■■□□□□□□□□□□□□■■■■■■■■■	36 (6.6)
	142	CAS1-Delhi	703777700003771	■■■□□□□■■■■■■■■■■■■■■□□□□□□□□□□□□□■■■■■■■■■	1 (0.2)
	357	CAS1-Delhi	703777740000771	■■■□□□□■■■■■■■■■■■■■■■□□□□□□□□□□□□□□■■■■■■■	4 (0.7)
	426	CAS	700377600003771	■■■□□□□□□□■■■■■■■■■■□□□□□□□□□□□□□□■■■■■■■■■	1 (0.2)
	428	CAS1-Delhi	703777740003371	■■■□□□□■■■■■■■■■■■■■■■□□□□□□□□□□□□■■□■■■■■■	2 (0.4)
	1343	CAS1-Delhi	703737740003771	■■■□□□□■■■■■□■■■■■■■■■□□□□□□□□□□□□■■■■■■■■■	2 (0.4)
	2145	CAS1-Delhi	703777640003771	■■■□□□□■■■■■■■■■■■■■□■□□□□□□□□□□□□■■■■■■■■■	1 (0.2)
	3391	CAS1-Delhi	703741000003771	■■■□□□□■■■■■■□□□□■□□□□□□□□□□□□□□□□■■■■■■■■■	1 (0.2)

	2373	CAS2	700337740003771	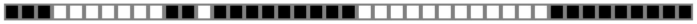	1 (0.2)
EAI (58)	11	EAI3-IND	477777777413071	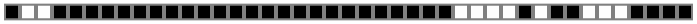	5 (0.9)
	126	EAI5	477777777413771	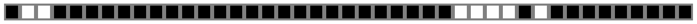	1 (0.2)
	138	EAI5	777777777413700	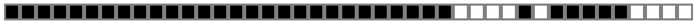	4 (0.7)
	19	EAI2-Man	677777477413771	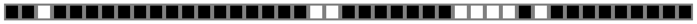	1 (0.2)
	27	EAI	703777747770371	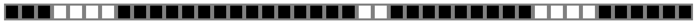	2 (0.4)
	236	EAI5	777777777413771	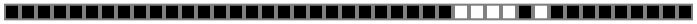	3 (0.6)
	292	EAI6-BGD1	777777757413371	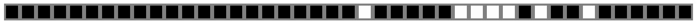	8 (1.5)
	337	EAI1- SOM	777777777013731	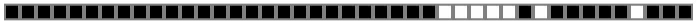	1 (0.2)
	470	EAI5	177777777413771	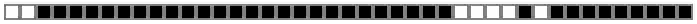	1 (0.2)
	48	EAI1- SOM	777777777413731	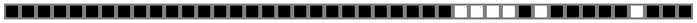	14 (2.6)
	591	EAI6-BGD1	777777757413771	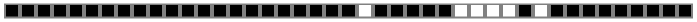	1 (0.2)
	882	EAI6-BGD	577777757413771	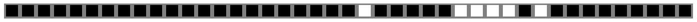	1 (0.2)
	1875	EAI5	477001777413771	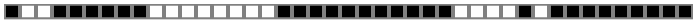	3 (0.6)
	234	EAI5	777777777413371	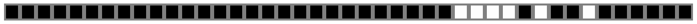	1 (0.2)
	1427	EAI5	457001777413771	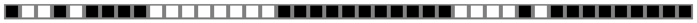	1 (0.2)

	1425	EAI6-BGD1	777777757413331	■■■■■■■■■■■■■■■■■■■■■■□■■■■■□□□□■□■■□■■□■■■	1 (0.2)
	1420	EAI6-BGD1	777777757410071	■■■■■■■■■■■■■■■■■■■■■■□■■■■■□□□□■□□□□□□■■■■	1 (0.2)
	1408	EAI5	000000007413371	□□□□□□□□□□□□□□□□□□□□□□□□■■■■□□□□■□■■□■■■■■■	1 (0.2)
	1391	EAI7-BGD2	777777700003371	■■■■■■■■■■■■■■■■■■■■■□□□□□□□□□□□□□■■□■■■■■■	1 (0.2)
	1389	EAI1-SOM	177777777413731	□□■■■■■■■■■■■■■■■■■■■■■■■■■■□□□□■□■■■■■□■■■	2 (0.4)
	2773	EAI6-BGD1	577775757413771	■□■■■■■■■■■■■■■■■□■■■■■□□□□■□■■■■■■■■■■	1 (0.2)
	3355	EAI1-SOM	777717777413731	■■■■■■■■■■■■□□■■■■■■■■■■■■■■□□□□■□■■■■■□■■■	2 (0.4)
	3274	EAI5	777777777413701	■■■■■■■■■■■■■■■■■■■■■■■■■■■■□□□□■□■■■■■□□□■	1 (0.2)
	1369	EAI5	477777777413671	■□□■■■■■■■■■■■■■■■■■■■■■■■■■□□□□■□■■■■□■■■■	1 (0.2)
	50	H3	777777777720771	■■■■■■■■■■■■■■■■■■■■■■■■■■■■■■□■□□□□■■■■■■■	2 (0.4)
H (5)	655	H3	477777777720771	■□□■■■■■■■■■■■■■■■■■■■■■■■■■■■□■□□□□■■■■■■■	2 (0.4)
	1159	H3	437777777720771	■□□□■■■■■■■■■■■■■■■■■■■■■■■■■■□■□□□□■■■■■■■	1 (0.2)
	42	LAM9	777777607760771	■■■■■■■■■■■■■■■■■■■■□□□□■■■■■■■■□□□□■■■■■■■	43 (7.9)
LAM (52)	61	LAM10-CAM	777777743760771	■■■■■■■■■■■■■■■■■■■■■■□□□■■■■■■■□□□□■■■■■■■	1 (0.2)
	64	LAM6	777777607560771	■■■■■■■■■■■■■■■■■■■■□□□□■■■■□■■■□□□□■■■■■■■	5 (0.9)
	150	LAM9	777767607760771	■■■■■■■■■■■■■■□■■■■■□□□□■■■■■■■■□□□□■■■■■■■	1 (0.2)

	1070	LAM9	777777607760371	■■■■■■■■■■■■■■■■■■■■□□□□■■■■■■■■□□□□□■■■■■■	1 (0.2)
	1933	LAM9	777777607760770	■■■■■■■■■■■■■■■■■■■■□□□□■■■■■■■■□□□□■■■■■■□	1 (0.2)
Manu (1)	54	Manu2	777777777763771	■■■■■■■■■■■■■■■■■■■■■■■■■■■■■■■■□□■■■■■■■■■	1 (0.2)
S (2)	34	S	776377777760771	■■■■■■■■□□■■■■■■■■■■■■■■■■■■■■■■□□□□■■■■■■■	2 (0.4)
T (93)	52	T2	777777777760731	■■■■■■■■■■■■■■■■■■■■■■■■■■■■■■■■□□□□■■■□■■■	1 (0.2)
	53	T1	777777777760771	■■■■■■■■■■■■■■■■■■■■■■■■■■■■■■■■□□□□■■■■■■■	38 (7.0)
	86	T1	777777737760771	■■■■■■■■■■■■■■■■■■■■■□■■■■■■■■■■□□□□■■■■■■■	1 (0.2)
	102	T	777703777760771	■■■■■■■■■■■■□□□□■■■■■■■■■■■■■■■■□□□□■■■■■■■	3 (0.6)
	123	T1	777777776360771	■■■■■■■■■■■■■■■■■■■■■■■■■■□□■■■■□□□□■■■■■■■	10 (1.8)
	240	T	777777777760371	■■■■■■■■■■■■■■■■■■■■■■■■■■■■■■■■□□□□□■■■■■■	3 (0.6)
	244	T1	777777777760601	■■■■■■■■■■■■■■■■■■■■■■■■■■■■■■■■□□□□■■□□□□■	19 (3.5)
	358	T1	717777777760771	■■■□□■■■■■■■■■■■■■■■■■■■■■■■■■■■□□□□■■■■■■■	17 (3.1)
	1105	T1	777773777760771	■■■■■■■■■■■■■■■□■■■■■■■■■■■■■■■■□□□□■■■■■■■	1 (0.2)
AFRI (1)	2819	AFRI_1	770777703017671	■■■■■■□□□■■■■■■■■■■■■□□□□■■□□□□□■■■■■■□■■■■	1 (0.2)
New (9)	2152	Undefined/New	777777702003371	■■■■■■■■■■■■■■■■■■■■■□□□□■□□□□□□□□■■□■■■■■■	8 (1.5)
	1089	Undefined/New	403777740003771	■□□□□□□■■■■■■■■■■■■■■■□□□□□□□□□□□□■■■■■■■■■	1 (0.2)

	NA	NA	477777777605471		2 (0.4)
	NA	NA	747777777740000		4 (0.7)
	NA	NA	476000017413071		2 (0.4)
	NA	NA	777777607620771		4 (0.7)
	NA	NA	747777777740001		2 (0.4)
	NA	NA	777777777412731		2 (0.4)
	NA	NA	400777774020771		1 (0.2)
	NA	NA	002002000001771		1 (0.2)
	NA	NA	777777742413371		1 (0.2)
	NA	NA	743777740000771		1 (0.2)
	NA	NA	717767434000771		1 (0.2)
	NA	NA	577747607760771		1 (0.2)
	NA	NA	777777743013771		1 (0.2)
	NA	NA	000017607760771		1 (0.2)
	NA	NA	477777757413371		1 (0.2)
	NA	NA	777677757413771		1 (0.2)

Orphan (66)	NA	NA	707777757013771	■■■□□□■■■■■■■■■■■■■■■■□■■■■□□□□□■□■■■■■■■■■	1 (0.2)
	NA	NA	703777774003371	■■■□□□□■■■■■■■■■■■■■■■■■■□□□□□□□□□■■□■■■■■■	1 (0.2)
	NA	NA	477777777013071	■□□■■■■■■■■■■■■■■■■■■■■■■□□□□□■□■■□□□■■■■	1 (0.2)
	NA	NA	457003777413771	■□□■□■■■■□□□□□□□■■■■■■■■■■■■□□□□■□■■■■■■■■■	1 (0.2)
	NA	NA	500003700003771	■□■□□□□□□□□□□□□□■■■■■□□□□□□□□□□□□□■■■■■■■■■	1 (0.2)
	NA	NA	740377740003771	■■■■□□□□□□■■■■■■■■■■■■□□□□□□□□□□□□■■■■■■■■■	1 (0.2)
	NA	NA	777777753003771	■■■■■■■■■■■■■■■■■■■■■■□■□■■□□□□□□□■■■■■■■■■	1 (0.2)
	NA	NA	777777702002771	■■■■■■■■■■■■■■■■■■■■■□□□□■□□□□□□□□■□■■■■■■■	1 (0.2)
	NA	NA	777777702003771	■■■■■■■■■■■■■■■■■■■■■□□□□■□□□□□□□□■■■■■■■■■	1 (0.2)
	NA	NA	777777777760401	■■■■■■■■■■■■■■■■■■■■■■■■■■■■■■■■□□□□■□□□□□■	1 (0.2)
	NA	NA	774777757413771	■■■■■■■□□■■■■■■■■■■■■■□■■■■■□□□□■□■■■■■■■■■	1 (0.2)
	NA	NA	777777757413770	■■■■■■■■■■■■■■■■■■■■■■□■■■■■□□□□■□■■■■■■■■□	1 (0.2)
	NA	NA	777777002000371	■■■■■■■■■■■■■■■■■■□□□□□□□■□□□□□□□□□□□■■■■■■	1 (0.2)
	NA	NA	717777777763771	■■■□□■■■■■■■■■■■■■■■■■■■■■■■■■■□□■■■■■■■■■	1 (0.2)
	NA	NA	777777742000071	■■■■■■■■■■■■■■■■■■■■■■□□□■□□□□□□□□□□□□□■■■■	1 (0.2)
	NA	NA	777777757413231	■■■■■■■■■■■■■■■■■■■■■■□■■■■■□□□□■□■■□■□□■■■	1 (0.2)

	NA	NA	763777400001771	■■■■■□□■■■■■■■■■■■■□□□□□□□□□□□□□□□□■■■■■■■■	1 (0.2)
	NA	NA	747777777760000	■■■■□□■■■■■■■■■■■■■■■■■■■■■■■■■■□□□□□□□□□□□	1 (0.2)
	NA	NA	777777777412771	■■■■■■■■■■■■■■■■■■■■■■■■■■■■□□□□■□■□■■■■■■■	1 (0.2)
	NA	NA	677777752402771	■■□■■■■■■■■■■■■■■■■■■■□■□■□■□□□□□□■□■■■■■■■	1 (0.2)
	NA	NA	743607400000771	■■■■□□□■■■■□□□□■■■■□□□□□□□□□□□□□□□□□■■■■■■■	1 (0.2)
	NA	NA	710000001013731	■■■□□■□□□□□□□□□□□□□□□□□□□□■□□□□□■□■■■■■□■■■	1 (0.2)
	NA	NA	740377600003771	■■■■□□□□□□■■■■■■■■■■□□□□□□□□□□□□□□■■■■■■■■■	1 (0.2)
	NA	NA	477777777413700	■□□■■■■■■■■■■■■■■■■■■■■■■■■■□□□□■□■■■■■□□□□	1 (0.2)
	NA	NA	777577757413371	■■■■■■■■■■□■■■■■■■■■■■□■■■■■□□□□■□■■□■■■■■■	1 (0.2)
	NA	NA	777777757413351	■■■■■■■■■■■■■■■■■■■■■■□■■■■■□□□□■□■■□■■■□■■	1 (0.2)
	NA	NA	777777702003271	■■■■■■■■■■■■■■■■■■■■■□□□□■□□□□□□□□■■□■□■■■■	1 (0.2)
	NA	NA	776177400000371	■■■■■■■■□□□■■■■■■■■□□□□□□□□□□□□□□□□□□■■■■■■	1 (0.2)
	NA	NA	777777607760730	■■■■■■■■■■■■■■■■■■■■□□□□■■■■■■■■□□□□■■■□■■□	1 (0.2)
	NA	NA	777707002013700	■■■■■■■■■■■■□□□■■■□□□□□□□■□□□□□□■□■■■■■□□□□	1 (0.2)
	NA	NA	717777777761771	■■■□□■■■■■■■■■■■■■■■■■■■■■■■■■■□□□■■■■■■■■	1 (0.2)
	NA	NA	777777777413500	■■■■■■■■■■■■■■■■■■■■■■■■■■■■□□□□■□■■■□■□□□□	1 (0.2)

	NA	NA	777777752003371	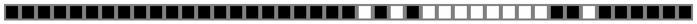	1 (0.2)
	NA	NA	700000000000071	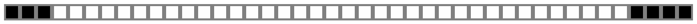	1 (0.2)
	NA	NA	777777702000000	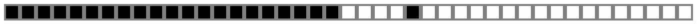	1 (0.2)
	NA	NA	703775000003771	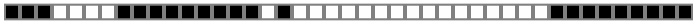	1 (0.2)
	NA	NA	757777702003351	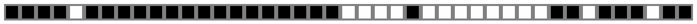	1 (0.2)
	NA	NA	177777737413731	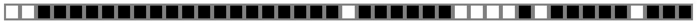	1 (0.2)
	NA	NA	703607400000771	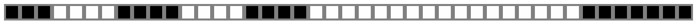	1 (0.2)
	NA	NA	703627400003771	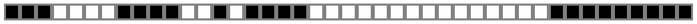	1 (0.2)

SIT, Spoligotype International Types assigned by SITVIT2WEB database; MDR-TB, multidrug resistant-tuberculosis; NA, not applicable (the SIT and lineages have not been assigned for Orphan isolates)
